# A Drier Maternal Environment Increases Water Stress Tolerance of Alpine Seeds and Seedlings

**DOI:** 10.1002/ece3.72247

**Published:** 2025-09-29

**Authors:** Jerónimo Vázquez‐Ramírez, Daniel J. White, Judd Harding, Megan J. Hirst, Tricia Wevill, Susanna E. Venn

**Affiliations:** ^1^ School of Life and Environmental Sciences Deakin University Melbourne Australia; ^2^ Royal Botanic Gardens Victoria Melbourne Australia

**Keywords:** Australian Alps, drought stress, germination, seed traits, seedling performance

## Abstract

The environmental conditions experienced by a mother plant during seed development can significantly influence the characteristics and performance of its offspring. These maternal environmental effects are crucial for understanding how plant species respond to climate variability and how they may be able to adapt in rapidly changing environments such as alpine ecosystems. While most studies in alpine environments have focused on the effects of warmer maternal temperatures, the consequences of reduced precipitation remain underexplored. We investigated the effects of a drier maternal environment on (i) seed size, (ii) germination and (iii) seedling water stress tolerance in three Australian alpine species (two forbs and one graminoid). We used rainout shelters to impose a 60% reduction in precipitation on maternal plants for 1 year. Then, seeds from plants in rainout and control plots were collected, measured for size and mass, and tested for germination under a gradient of water potential solutions (0 to −1.0 MPa using PEG 6000). Seedlings were grown and subjected to a gradient of watering treatments (100%, 80% and 60% pot capacity) for 14 days under controlled conditions. A drier maternal environment affected seed and seedling traits in all three species, with life‐form and species‐specific responses. Seed mass and size decreased in the two forbs but increased in the graminoid. In general, seeds collected from rainout shelters had higher germination under severe water stress (−1.0 MPa). Seedlings from drier maternal environments generally exhibited larger total leaf area and lower physiological stress under severe water stress (60% pot capacity). Our findings demonstrate that reduced precipitation during seed development can enhance offspring drought tolerance in alpine species, particularly under severe stress. These maternal effects may contribute to short‐term adaptive responses to climate change by increasing offspring performance under water‐limited conditions.

## Graphical abstract

We investigated the effects of a drier maternal environment on (i) seed morphology, (ii) germination and (iii) seedling water stress tolerance in three alpine species. Overall, our results showed that a drier maternal environment affected seed and seedling traits in all three species, with life‐form and species‐specific responses. In general, seeds and seedlings from a drier maternal environment have better tolerance to water stress.

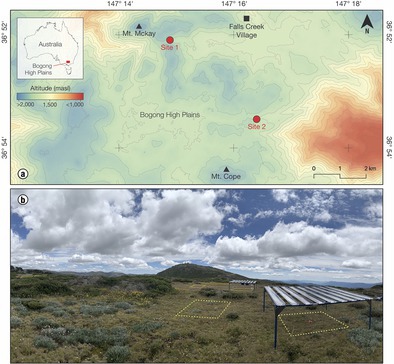

## 1 Introduction

Maternal environmental effects refer to the influence of the environment experienced by a mother plant on the phenotype and performance of its offspring (Donohue 2009). These effects are driven by environmental factors such as temperature, water availability, nutrient levels and stressors such as extreme temperatures or drought (Donohue 2009; Nicotra et al. 2010). They result from physiological adaptations in the mother plant that affect resource allocation, hormonal signalling or epigenetic modifications, and can occur at different stages of sexual reproduction (Herman and Sultan 2016; Münzbergová et al. 2019; Penfield and MacGregor 2017). This mechanism allows plants to ‘carry over’ information about the maternal environment, influencing the ability of their offspring to establish and cope with stress (Donohue 2009).

Maternal environmental effects play an important role in enabling plants to adapt to changing environmental conditions, including those associated with climate change (Nicotra et al. 2010). This type of phenotypic plasticity can influence key traits such as seed dormancy, germination and seedling stress tolerance, all of which are essential for plant establishment and survival in rapidly changing environments such as alpine ecosystems (Fenner 1991; Fernández‐Pascual et al. 2019; Penfield and MacGregor 2017). In these cold‐climate regions, maternal effects may help plant species buffer against changing environmental conditions, such as warmer temperatures, changes in snow cover and shifts in soil moisture regimes.

Maternal environmental effects on alpine seeds and seedlings have been studied previously, with particular emphasis on the effects of warmer maternal temperatures (e.g., Arnold et al. 2024; Bernareggi et al. 2016; Nicotra et al. 2015; Notarnicola et al. 2023; Veselá et al. 2021; Wang et al. 2021). For example, in an Australian alpine forb, warmer maternal temperatures led to reduced seed mass and a higher proportion of dormant seeds (Notarnicola et al. 2023). Similarly, in the Italian Alps, warmer maternal temperatures increased seed germinability following cold stratification in three snowbed forb species but had no effect on germination time (Bernareggi et al. 2016). In two Norwegian alpine forbs, maternal environmental conditions influenced both total germination percentage and germination time (Veselá et al. 2021). These findings highlight the significant role of maternal environmental conditions in shaping alpine plant recruitment traits.

However, temperature is not the only environmental factor changing in alpine regions; decreases in precipitation are also projected for areas such as the southern Andes, the European Mediterranean mountains and the Australian Alps (Hock et al. 2019). This is particularly important, as the early life‐history stages of plants are highly sensitive to water deficits, such that the net effects of warming temperatures on plant recruitment may largely depend on soil moisture (Mondoni et al. 2022; Vázquez‐Ramírez and Venn 2021).

Water availability in the maternal environment can influence both morphological and physiological traits related to offspring performance. Existing studies have reported changes in seed mass (mainly reduction) and dormancy under drier maternal conditions (e.g., Espinosa del Alba et al. 2025; Gya et al. 2023; Peng et al. 2020; Rosbakh et al. 2017). In terms of germination, drier maternal environments can enhance the ability of seeds to germinate under water stress. For example, in two Arctic‐alpine forbs, seeds from plants growing on drier sites had higher germination rates under low water potential solutions than those from wetter sites (Gya et al. 2023). Similarly, seeds of a Mediterranean‐alpine sub‐shrub from drier microclimates had a lower base water potential for germination compared to those from wetter sites (Espinosa del Alba et al. 2025). At the seedling stage, maternal drier conditions have also been associated with shifts in biomass allocation and improved performance under water stress in Arctic‐alpine species (Gya et al. 2023).

Despite the importance of water availability for alpine plant recruitment, and the fact that projected reductions in precipitation will coincide with critical stages of the plant reproductive cycle, our understanding of how these changes interact with maternal effects remains limited. For example, in the Australian Alps, more frequent and intense soil water deficits are predicted to occur in summer as a consequence of reduced precipitation and warmer temperatures (Sánchez‐Bayo and Green 2013), at a time when most plant species are producing seeds (Costin et al. 2000). The potential consequences of reduced water availability during seed development remain unclear; specifically, how this stress will affect seeds and seedlings via maternal effects, and to what extent these effects facilitate adaptation to drier conditions.

Here, we investigated the effects of a drier maternal environment on (i) seed size, (ii) germinability across a gradient of water availability and (iii) seedling water stress tolerance (morphological and physiological responses) in three Australian alpine species. To do so, we manipulated maternal water availability in situ for 14 months using rainout shelters, and then conducted germination and seedling experiments under controlled laboratory conditions. By integrating responses across multiple early life‐history stages, we aimed to improve our understanding of the resilience of alpine plants to drier climate projections and to inform evidence‐based strategies for the conservation and restoration of these ecosystems under climate change.

## 2 Materials and Methods

### 2.1 Study Area and Study Species

**FIGURE 1:**
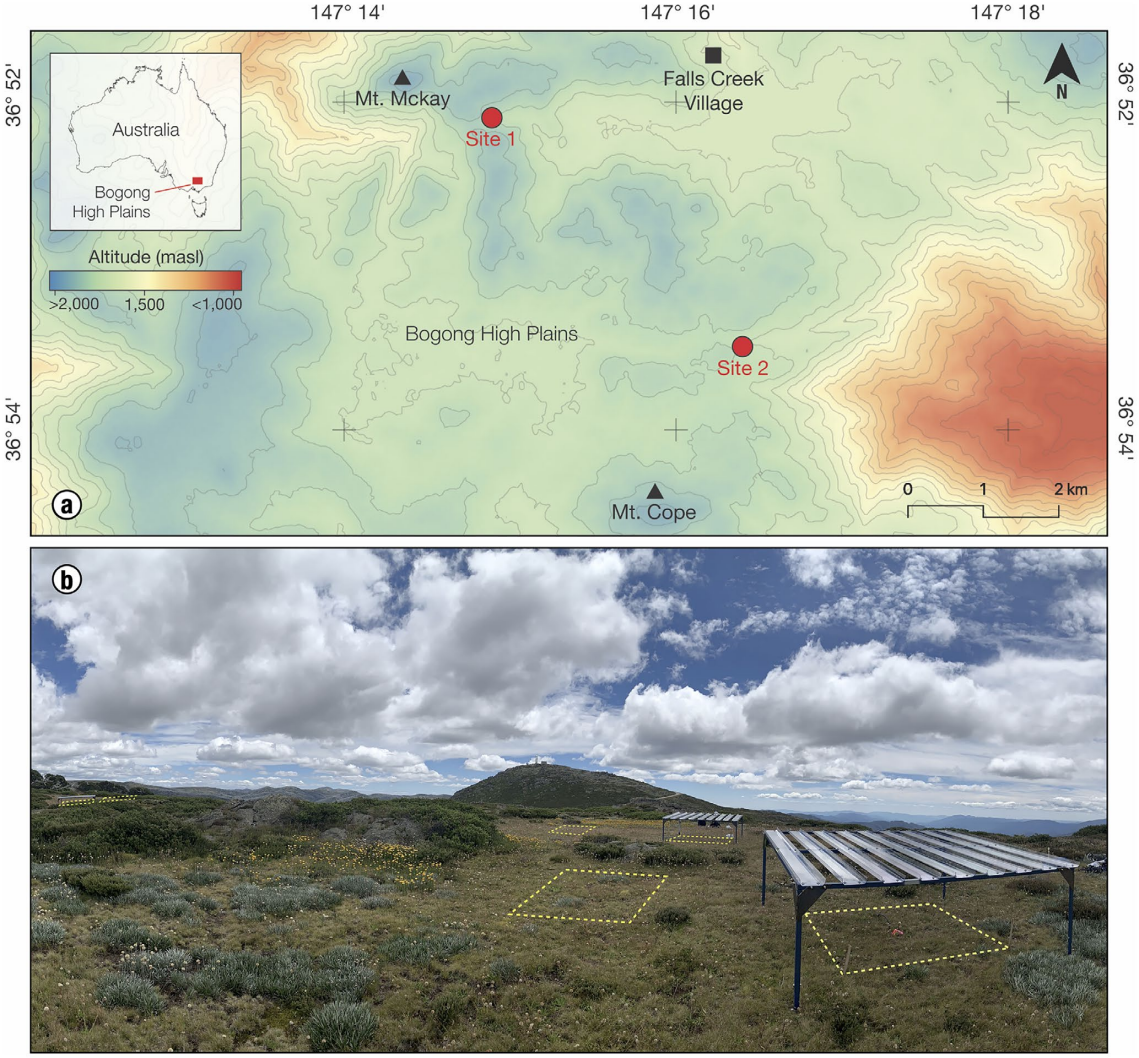
(a) Location of the two study sites (red labelled dots) within the Bogong High Plains, Victoria, Australia. (b) Photograph showing the experimental design at each site.

The field phase of the study was conducted on the Bogong High Plains, located in the Victorian Alps, south‐eastern Australia (Figure 1). The mean annual temperature is 9.5°C (1990–2021). The mean annual precipitation is 1307 mm (1990–2021), with most of the precipitation falling as snow during the winter and the driest period of the year being summer (Bureau of Meteorology 2022). Soils in the region are free draining, highly acidic alpine humus (Costin et al. 2000). The study was conducted in a tall alpine herbfield, the most common and widespread alpine plant community in Australia (Costin et al. 2000).

We studied three native alpine plant species: *Plantago euryphylla* B.G.Briggs, Carolin & Pulley (Plantaginaceae), *Pappochroma bellidioides* (Hook.f.) G.L.Nesom (Asteraceae) and *Rytidosperma nudiflorum* (P.Morris) Connor & Edgar (Poaceae). The species were selected based on their abundance within the experimental plots (see Section 2.2). All species flower in early spring and disperse seeds in late summer. All three species use the C3 photosynthetic pathway, in line with their alpine ecology and distribution.

### 2.2 Rainout Shelters and Seed Collection

Two sites within the study area were selected (Site 1: 36.876730° S, 147.249689° E, 1765 m a.s.l.; and Site 2: 36.909121° S, 147.297240° E, 1670 m a.s.l.) and at each site, three pairs of 2 × 2 m plots with similar plant species composition and topographic conditions were established (Figure 1a). Plots within each pair were separated by < 3 m, and pairs were located within 10 m of each other. In February 2021, one plot from each pair was covered with a rainout shelter and the remaining plots were left under ambient conditions and served as controls (Figure 1b). The rainout shelters measured 2.5 × 2.5 m, with a height of 1.3 m at the highest point and 1.0 m at the lowest, and 60% of their surface was covered by transparent, weatherproof polycarbonate strips set at a 10% slope to divert intercepted rainfall away from the experimental plots. To assess the impact of the shelters on soil moisture, we installed a HOBO‐S‐SMD‐M005 moisture sensor in the centre of each plot (see https://www.amrf.org.au). Although we did not measure temperature, light or ambient relative humidity under the rainout shelters, previous studies looking at the performance of similar shelters have found only minor or negligible effects on these and other environmental variables (Kundel et al. 2018; Yahdjian and Sala 2002).

In April 2022, 14 months after the installation of the rainout shelters, we collected seeds from > 20 individuals of each species in both maternal environments (i.e., control and sheltered plots). Seed collection for each species was conducted at a single site to ensure consistency in population origin: *P. euryphylla* and *P. bellidioides* were collected at site 1, and *R. nudiflorum* at site 2 (Figure 1). Seeds were pooled by maternal environment, placed in paper bags immediately after collection and transported to the laboratory, where they were stored at ambient temperature for approximately 2.5 months before the start of the experiments.

### 2.3 Seed Size and Germination Across a Gradient of Water Potential Solutions

In the laboratory, seeds were cleaned and their dispersal structures removed. For each species and maternal environment, we determined seed mass using five replicate lots of 100 seeds. To measure seed length, we photographed each weighed lot and used ImageJ software to measure the longest axis of 10 seeds per replicate.

To test whether a drier maternal environment improves germinability under water stress, we conducted germination trials across a gradient of water potential solutions (0, −0.25, −0.5, −0.75, −1.0 MPa). We prepared the solutions using deionised water and polyethylene glycol (PEG 6000; Merck), a high‐molecular‐weight polymer commonly used in germination studies to simulate drought stress because it cannot cross plant cell walls (Emmerich and Hardegree 1990). We followed the method of Kaufmann and Michel (1973) to prepare the solutions. For each combination of maternal environment and water potential, we used five replicates containing 10 seeds for *R. nudiflorum* and 20 seeds for *P. euryphylla* and *P. bellidioides*, based on seed availability. We placed the seeds in 90 mm transparent plastic Petri dishes with two sheets of Advantec Grade No. 101 germination filter paper soaked in the designated PEG solution. To minimise changes in water potential due to PEG retention by the paper or evaporation, we added 8 mL of solution to each dish (solution‐to‐paper ratio of 14) and sealed the dishes with parafilm (Emmerich and Hardegree 1991). We then placed the dishes in a CLIMATRON‐520‐DL growth chamber set to the optimum germination temperature of 20°C for Australian alpine species (Hirst et al. 2025) under a 14‐h light (3500 lx) and 10‐h dark cycle. We used a constant temperature because PEG solutions are temperature dependent, and alternating day/night temperatures could potentially alter their initial osmotic weight.

Germination was recorded every 2–3 days for 30 days. Radicle emergence (> 1 mm) was the criterion for recording a seed as germinated. To assess the viability of non‐germinated seeds, we performed a cut test and classified seeds with firm, white endosperm as potentially viable. We excluded empty seeds from the initial counts. We decided not to subject our experimental seeds to cold stratification as our germination test was carried out shortly after seed collection and, based on our experience with the study species, they are able to germinate without it.

### 2.4 Seedlings Water Stress Tolerance

We used a subset of the collected seeds to grow 30 seedlings per species per maternal environment (60 seedlings per species, 180 seedlings in total). We germinated seeds in 78 mL black plastic pots filled with Scotts Osmocote Native Premium Mix, a commercial substrate formulated for Australian native plants. Pots were placed in a greenhouse under well‐watered conditions (100% pot capacity; see below).

To determine pot capacity, we first weighed the empty pots filled with dry soil. We then irrigated the pots to saturation, allowed them to drain for 4 h at room temperature in a shaded area, and recorded their saturated weight. The difference between dry and saturated weight was used to calculate the soil water holding capacity (hereafter referred to as pot capacity).

Once the seedlings developed two pairs of true leaves (or four leaves in the case of *R. nudiflorum* ), we assigned them to three water stress treatments: 100% pot capacity (no stress), 80% (moderate stress) and 60% (severe stress). These treatments represent a controlled gradient of water stress. Although not directly aligned with field soil moisture levels, they were selected to impose a consistent and reproducible range of stress across species and maternal environments. Each treatment group contained 10 plants per species × maternal environment combination. We applied the treatments gradually over 1 week by reducing watering, then transferred seedlings to a growth chamber (CLIMATRON‐520‐DL) set to 20/10°C with a 14‐h light (3500 lx) and 10‐h dark cycle. We maintained temperature, light and watering conditions for 14 days. To ensure target pot capacity, we weighed each pot every 2–3 days and added water to maintain pre‐determined weights.

After 2 weeks, we measured total leaf area (TLA), relative water content (RWC) and chlorophyll fluorescence (*F*_V_/*F*_M_) to assess the morphological and physiological responses of the experimental seedlings to water stress. We focused on these traits due to their relevance to early seedling establishment and drought response. TLA reflects early biomass allocation and photosynthetic capacity of an individual plant; RWC provides insight into plant water status and tissue hydration (Barrs and Weatherley 1962); and *F*_V_/*F*_M_ is a widely used indicator of photochemical efficiency and physiological stress in response to drought (Maxwell and Johnson 2000).

To estimate TLA per seedling, we counted the number of leaves and multiplied by the average leaf area for the respective species and treatment. We calculated average leaf area by collecting one fully developed and representative leaf per plant, measuring its surface area using ImageJ software and averaging values across each treatment group.

To calculate RWC, we randomly selected five plants per species and treatment, and collected three fully expanded leaves per plant. We recorded their fresh weight, then submerged them in distilled water in dark conditions for 3 h at room temperature to determine turgid weight. We then dried the leaves at 80°C for 5 days and recorded dry weight. We calculated RWC as:

$$\mathrm{RWC}=\left[\left(\text{fresh weight}-\mathrm{dry}\;\text{weight}\right)/\left(\text{turgid weight}-\mathrm{dry}\;\text{weight}\right)\right]\times 100$$

Chlorophyll fluorescence (*F*_V_/*F*_M_; maximum quantum efficiency of open PSII centres) was measured weekly (on Days 0, 7 and 14) using five randomly selected plants per species per maternal environment × watering treatment combination. We marked these plants at the start of the experiment, while keeping the rest as backups in case of mortality. For each plant, we took three fluorescence readings on each of three leaves and used the average of the nine values to obtain a single *F*_V_/*F*_M_ ratio per plant per time point. Measurements were made using an Imaging PAM (Model IMAG‐MIN/B; Walz, Effeltrich, Germany). Prior to measurement, seedlings were dark‐adapted for 60 min to ensure accurate readings and followed the recommendations of Maxwell and Johnson (2000).

### 2.5 Data Analysis

We conducted all statistical analyses in R (R Core Team 2022) and created figures using the ‘ggplot2’ package (Wickham 2016), with minor visual edits performed in Adobe Illustrator. Analyses were performed separately for each study species and consisted of four components:

#### 2.5.1 Soil Moisture

To test for differences in soil volumetric water content (m^3^/m^3^) between treatments, we fitted a robust Linear Mixed Model (LMM) using the ‘robustlmm’ package (Koller 2016). We included mean daily volumetric water content as the response variable and treatment (shelter vs. control) as a fixed effect, and site was included as a random intercept to account for spatial replication. Initial diagnostics using standard linear mixed models revealed violations of the assumptions of normality and homoscedasticity, justifying the use of a robust modelling approach. Model validation was based on a visual inspection of residual and *Q*‐*Q* plots, which confirmed that the robust model had adequately down‐weighted influential observations and improved the behaviour of the residuals overall.

#### 2.5.2 Seed Size

To assess whether seed size differed between maternal environments, we fitted linear models. We used 100‐seed mass (g) or seed length (cm) as the response variable, with treatment as a fixed effect. Where necessary, we log‐transformed response variables to meet assumptions of normality and homoscedasticity. All models were fitted using base R functions, and we assessed assumptions through visual inspection of residual plots. Due to the small sample size and low residual variance, some diagnostic plots exhibited numerical instability. However, there were no systematic deviations in the residuals and fitted values, and the model fit was otherwise adequate.

#### 2.5.3 Germination

We calculated final percentage germination (FPG) and mean germination rate (MGR) using the ‘GerminaR’ package (Lozano‐Isla et al. 2019).

Then, to determine whether seeds from different maternal environments showed overall differences in FPG and MGR response across the water potential gradient, we fitted generalised additive mixed models (GAMMs) using the package ‘mgcv’ (Wood and Scheipl 2014). These models accounted for the non‐linear relationship often observed between germination and water potential. In the models, FPG (binomial model) and MGR (Gaussian model) were used as response variables, with water potential included as a continuous predictor fitted with a smoothing term. We included an interaction between water potential and maternal environment to allow maternal environment‐specific curves. Petri dish was added as a random intercept. We compared the full model (with interaction) to a reduced model (without interaction) using likelihood ratio tests based on the log‐likelihood of the mixed‐effects components.

To assess differences in FPG and MGR at specific water potential levels, we fitted additional linear mixed models (LMMs) and generalised linear mixed models (GLMMs) using the ‘glmmTMB’ package (Brooks et al. 2017). In the models, FPG (binomial model) or MGR (gaussian model) was included as a response variable, with water potential, maternal environment and their interaction as fixed effects. The Petri dish was included as a random intercept. Post hoc pairwise comparisons between maternal environments at each water potential level were conducted using the ‘emmeans’ package (Lenth 2023).

#### 2.5.4 Seedlings

To determine differences in morphological and physiological traits across maternal environments and watering treatments at the end of the experiment (Day 14), we fitted separate linear models for each trait. In these models, total leaf area (TLA), relative water content (RWC) or *F*_V_/*F*_M_ was included as response variable, with maternal environment, watering treatment and their interaction specified as fixed effects. We performed post hoc pairwise comparisons between maternal environments at each watering treatment level, adjusting *p*‐values using the Tukey method.

To assess whether *F*_V_/*F*_M_ trajectories differed between maternal environments over time (because we measured it on 0, 7 and 14 days), we fitted GAMMs as the exploratory analysis showed a non‐linear relationship. In the models, *F*_V_/*F*_M_ was the response variable and time was included as a continuous predictor with a smoothing term. Maternal environment was specified as a fixed effect, and we included an interaction between maternal environment and the smoother to allow maternal environment‐specific curves. Individual plant identity was included as a random intercept to account for repeated measures. Separate models were fitted for each watering treatment (100%, 80% and 60% pot capacity). For each treatment, we compared the full model (with environment‐specific smooths) to a reduced model with a single smooth term shared across conditions using likelihood ratio tests. Then, to examine differences in *F*_V_/*F*_M_ between maternal environments at specific time points, we performed pairwise comparisons on Days 0, 7 and 14.

## 3 Results

### 3.1 Environmental Conditions in the Field

The mean annual temperature during the field phase (2021–2022) was 9.6°C, similar to the historical average for the region (9.5°C). In contrast, total annual precipitation during the study was above average (1900 mm vs. 1336 mm for 1990–2024; Bureau of Meteorology, www.bom.gov.au). Although sensor malfunctions prevented us from recording soil moisture for the entire study period, the available data confirm that rainout shelters significantly reduced soil moisture during the reproductive period of the study species. On average, shelter plots reduced volumetric water content by ~5% compared to control plots (Table S1, Figure S1). While soil moisture was significantly reduced under rainout shelters, ambient relative humidity may have mitigated the extent of physiological water stress by reducing transpiration demand, particularly since the study year was wetter than average. However, ambient relative humidity was not measured in this study, and this is acknowledged as a limitation.

### 3.2 Seed Size

**FIGURE 2:**
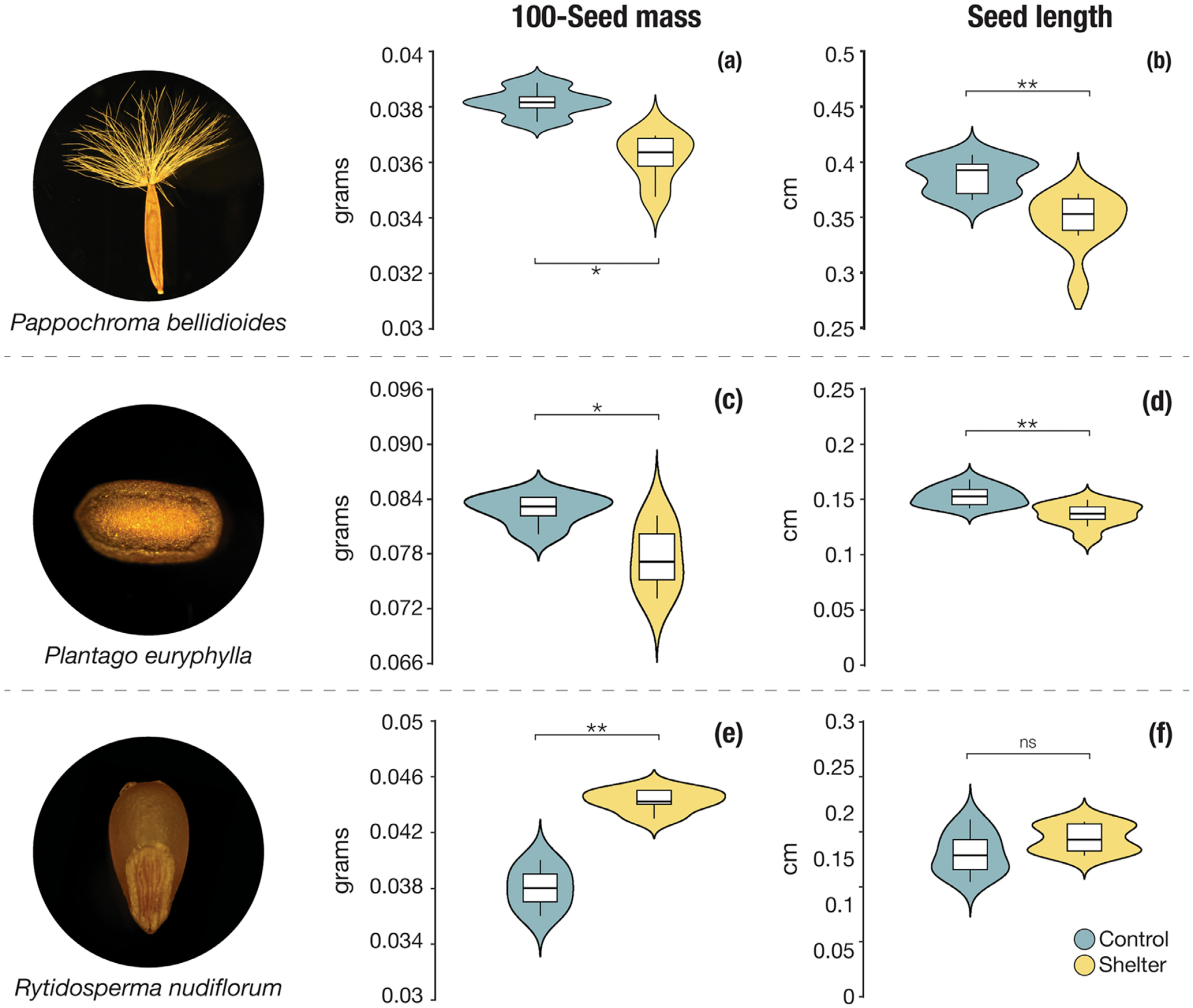
Effects of a 1‐year rainout shelter treatment on seed mass (a, c, e) and seed length (b, d, f) in the study species. Asterisks indicate significance levels: *p < 0.05; **p < 0.01; ns, not significant. See Table S2 for model outputs.

The maternal environment significantly influenced seed size. In *P. euryphylla* and *P. bellidioides*, seeds produced under drier conditions (i.e., shelter plots) were approximately 5% lighter and 10% shorter in length compared to those from control plots. In contrast, *R. nudiflorum* produced seeds that were 16% heavier in shelter plots, with no significant change in seed length (Table S2, Figure 2).

### 3.3 Germination

**FIGURE 3:**
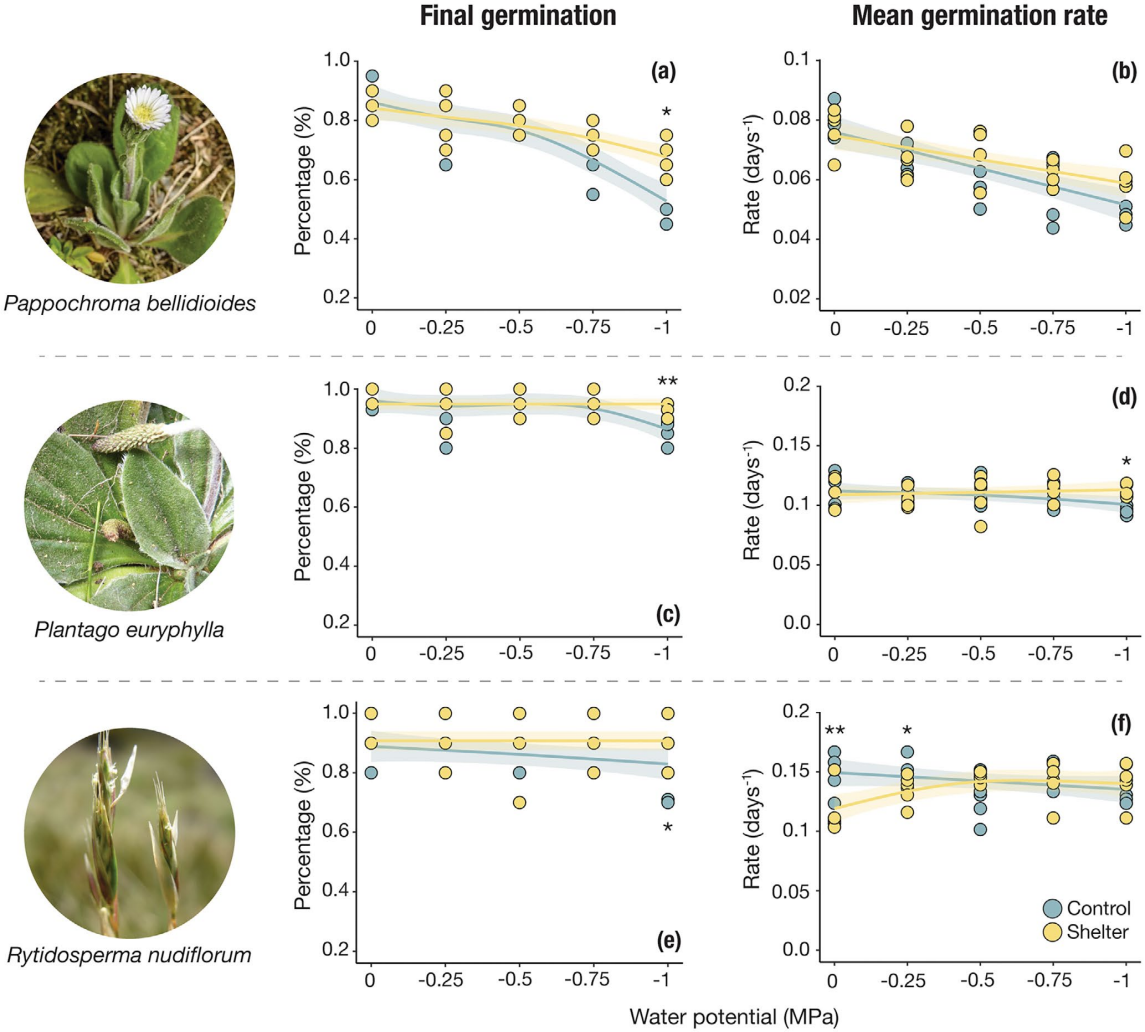
Effects of water potential solutions on final germination percentage (a, c, e) and mean germination rate (b, d, f) for seeds from different maternal environments. Lines show model predictions, and shaded areas represent 95% confidence intervals. Asterisks indicate significance levels from pairwise comparisons: *p < 0.05; **p < 0.01. See Table S5 for full model outputs.

Final percentage germination declined with decreasing water potential in all species, but the strength of the decline and the influence of maternal environment were species‐specific (Figure 3). In *P. euryphylla*, germination responses across the gradient of water potential solutions (i.e., GAMM curves) differed between control and shelter seeds (LRT: *χ* ^2^ = 6.85, *p* = 0.033). In contrast, *P. bellidioides* and *R. nudiflorum* showed similar overall curve shapes between maternal environments (LRT: *χ* ^2^ = 3.13, *p* = 0.29; *χ* ^2^ = 4.60, *p* = 0.11, respectively), although shelter seeds tended to exhibit less‐steep declines in germination at lower water potentials (Table S3).

GLMMs revealed significant main effects of both water potential and maternal environment across all species (Table S4), but detected no interaction. Pairwise comparisons indicated that shelter seeds germinated significantly more than control seeds, but only at the most negative water potential (−1.0 MPa; Table S5, Figure 3).

Mean germination rate also varied with water potential and maternal environment, again in a species‐specific manner. In *P. bellidioides*, MGR declined consistently with increasing drought severity under both maternal environments. In contrast, *R. nudiflorum* and *P. euryphylla* displayed more stable MGR values across the water potential gradient, particularly in shelter seeds.

There were no significant differences in the overall responses of MGR across the gradient of water potential solutions between maternal environments (LRTs: *χ* ^2^ = 5.30, *p* = 0.071 for *R. nudiflorum* ; *χ* ^2^ = 1.74, *p* = 0.418 for *P. euryphylla*; *χ* ^2^ = 0.42, *p* = 0.81 for *P. bellidioides*). However, GLMMs and post hoc comparisons (Tables S4 and S5) revealed that *R. nudiflorum* shelter seeds had significantly lower MGR than control seeds at 0 and −0.25 MPa, while *P. euryphylla* shelter seeds had significantly higher MGR at −1.0 MPa. No significant differences in MGR were observed between maternal environments in *P. bellidioides*.

### 3.4 Seedling Water Stress Tolerance

**FIGURE 4:**
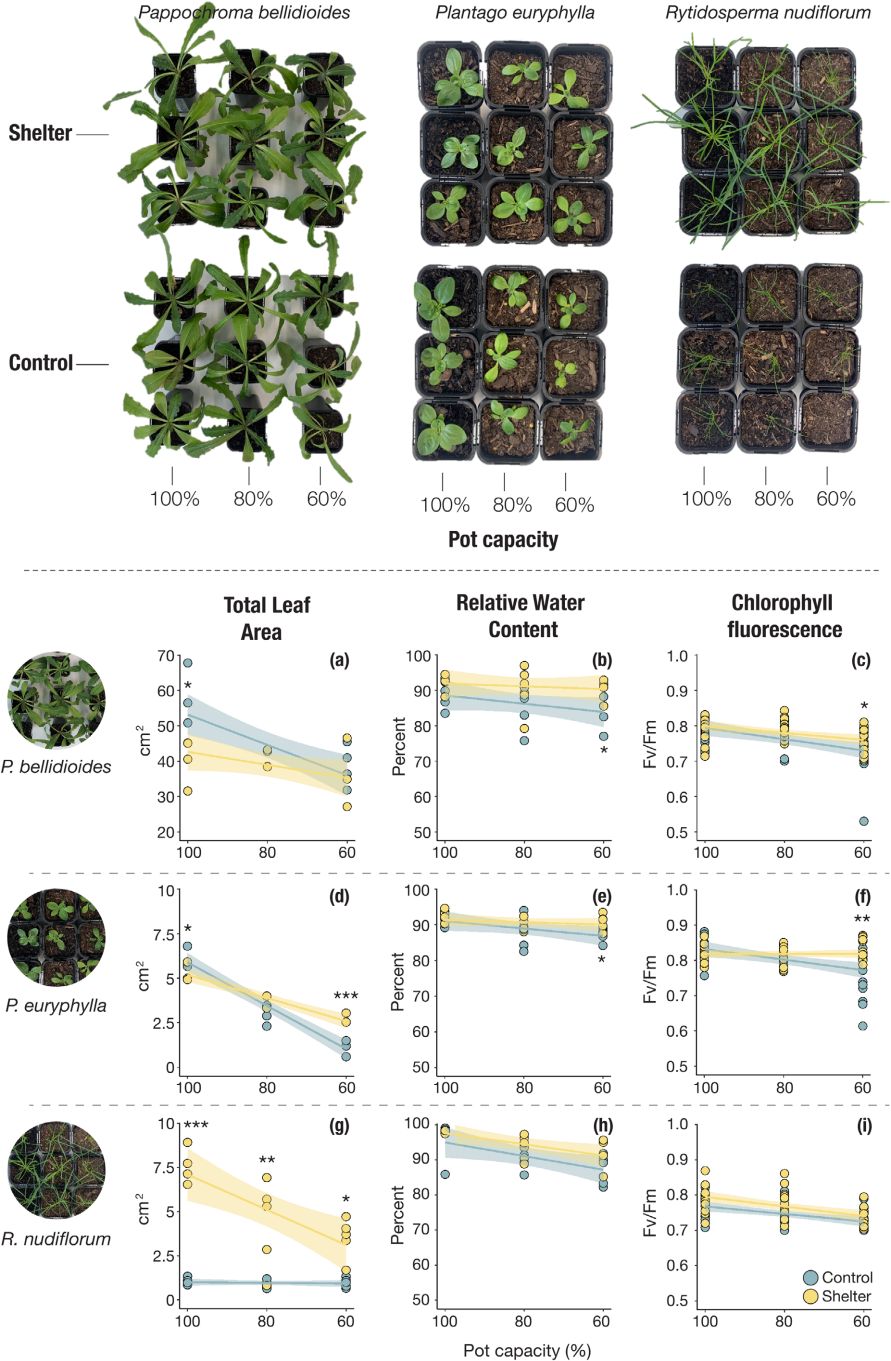
Total leaf area (a–c), relative water content (d–f) and chlorophyll fluorescence (g–i) of seedlings after 14 days of watering treatments in the climate chamber experiment. Lines show model predictions, and shaded areas represent 95% confidence intervals. Asterisks indicate significance levels from statistical models: *p < 0.05; **p < 0.01; *** *p* < 0.001. See Tables S6 and S7 for model and post hoc results.

Total leaf area declined with increasing water stress across all species and maternal environments (Table S6, Figure 4). Under well‐watered conditions (100% pot capacity), seedlings from larger seeds (i.e., control maternal environment) had greater TLA. However, this trend was reversed under stress: at 60% pot capacity, seedlings from drier maternal environments (regardless of size) had significantly greater TLA, with significant effects in *P. euryphylla* and *R. nudiflorum* (Table S7). In the latter, this positive maternal effect was consistent across all watering treatments, with sheltered seedlings consistently outperforming those from control conditions (Figure 4).

Relative water content (RWC) also declined with increasing water stress, with the lowest values recorded at 60% pot capacity (Table S6, Figure 4). Seedlings from drier maternal environments generally maintained higher RWC than those from control environments, although this effect was statistically significant only under severe water stress for *P. bellidioides* and *P. euryphylla* (Table S7, Figure 4). Among species, *R. nudiflorum* had the highest overall RWC range (85%–99%), followed by *P. euryphylla* (80%–95%) and *P. bellidioides* (75%–95%). Chlorophyll fluorescence (*F*_V_/*F*_M_) values at the end of the experiment followed the same pattern (Table S7, Figure 4).

**FIGURE 5:**
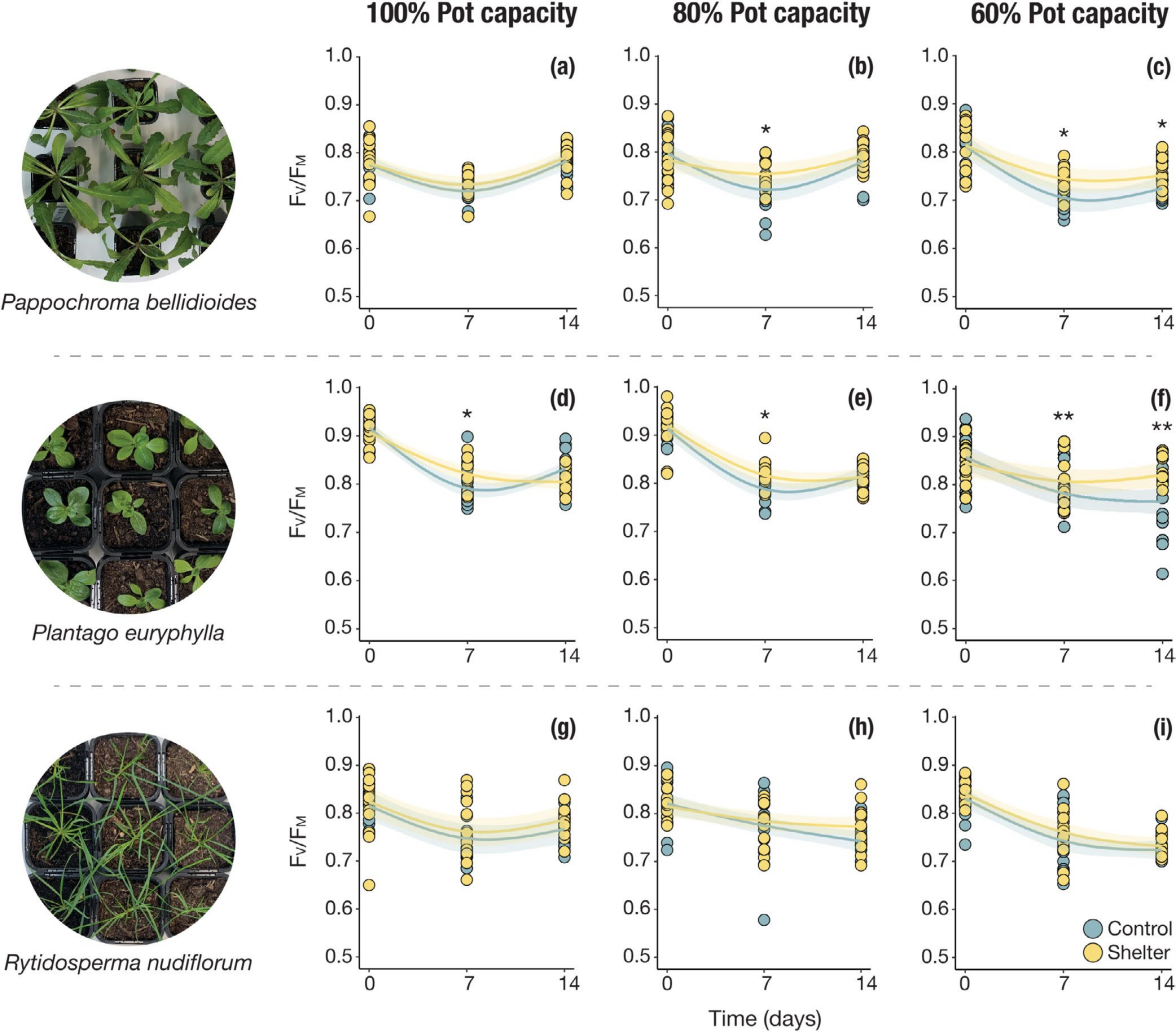
Weekly chlorophyll fluorescence (*F*_V_/*F*_M_) values across species and watering treatments. Lines show model predictions, and shaded areas represent 95% confidence intervals. Asterisks indicate significance levels from statistical models: *p < 0.05; **p < 0.01. See Tables S8 and S9 for model and post hoc results.

Weekly *F*_V_/*F*_M_ values showed non‐linear trajectories over time, driven by a marked decline in values measured at Day 7, likely reflecting an initial stress response to the growth chamber environment (Figure 5). At this time point, significant differences between maternal environments were observed for *P. bellidioides* and *P. euryphylla*, with shelter seedlings exhibiting higher *F*_V_/*F*_M_ values (Table S8). By Day 14, plants from different maternal environments under 100% and 80% pot capacity showed no significant differences in chlorophyll fluorescence. However, at 60% pot capacity, *F*_V_/*F*_M_ values remained low, and seedlings from drier maternal environments consistently outperformed those from control plots in both *P. bellidioides* and *P. euryphylla*. In *R. nudiflorum* , this pattern was also evident, but no significant differences between treatments were observed at any time point (Table S8).

Likelihood ratio tests comparing full and reduced models showed that maternal environment significantly influenced *F*_V_/*F*_M_ curves in *P. euryphylla*, but not in *P. bellidioides* or *R. nudiflorum* under any watering treatment (Table S9).

## 4 Discussion

Our results show that, despite some species‐specific responses at particular life stages, a drier maternal environment enhances seed and seedling tolerance to water stress. Across species, seeds and seedlings from the drier maternal environment generally exhibited higher germination success, greater total leaf area, higher relative water content and improved photosynthetic efficiency under severe water limitation (−1.0 MPa and 60% pot capacity). These results suggest that maternal environmental effects may help alpine species to tolerate and buffer against climate change and expected shifts in precipitation regimes (Hock et al. 2019).

### 4.1 Seed Traits

The reduction in seed mass and size as a consequence of a drier maternal environment observed in *P. euryphylla* and *P. bellidioides* is consistent with patterns reported for other Arctic‐alpine species, both in manipulative experiments and along natural moisture gradients (Peng et al. 2020; Rosbakh et al. 2017). In contrast, the increase in seed mass observed in *R. nudiflorum* under the same conditions is more similar to the response of some alpine species to warmer maternal temperatures (e.g., Cui et al. 2017; Totland and Alatalo 2002; Wirth et al. 2010).

From an ecological perspective, seed mass is linked to other seed traits, such as dispersal potential and persistence in soil seed banks (Saatkamp et al. 2019). The contrasting responses between species may reflect different strategies for coping with maternal drought stress, which may also have important implications for recruitment. Smaller, lighter seeds are better suited to long‐distance dispersal, potentially allowing them to escape drier microsites—a crucial advantage given the generally low dispersal capacity of alpine species (Morgan and Venn 2017). However, their smaller size makes them more likely to be buried deeper in the soil (Thompson et al. 1993), which may limit germination, as light is essential for many alpine species to germinate (Fernández‐Pascual et al. 2021). In contrast, heavier seeds remain closer to the parent plant but carry more reserves to support seedling establishment under local drought conditions and are less likely to be buried into the soil profile.

A limitation of our study is that we did not assess reproductive effort (e.g., seed number or seeds per fruit) across maternal environments. This prevented us from exploring potential trade‐offs between seed mass and seed number, relationships that are known to be influenced by maternal effects (e.g., Notarnicola et al. 2023) and may have helped to explain the contrasting species‐specific responses we found. We recommend that future research investigate these trade‐offs across a gradient of water availability to better understand how a drier maternal environment influences reproductive allocation strategies in alpine plants.

### 4.2 Germination

Seeds from drier maternal environments exhibited significantly higher germination (FPG) under the most negative water potential tested (−1.0 MPa), suggesting an adaptive advantage under severe drought. Similar results have been reported in alpine plants growing under drier conditions in both Arctic‐alpine (Gya et al. 2023; Veselá et al. 2021) and Mediterranean‐alpine (Espinosa del Alba et al. 2025) environments. Interestingly, despite the field phase of our experiment coinciding with a wetter‐than‐average year, seeds from control conditions germinated well under moderate water stress (−0.2 to −0.8 MPa). This suggests that the study species, particularly *P. euryphylla* and *R. nudiflorum* , are inherently tolerant to water limitation during germination. Our findings contrast with those reported for two arcto‐alpine forbs (Gya et al. 2023), a Mediterranean‐alpine sub‐shrub (Espinosa del Alba et al. 2025) and six alpine species in the Italian Alps (Orsenigo et al. 2015), where germination declines sharply around −0.75 MPa.

Drier maternal conditions also affected germination speed (MGR) in species‐specific ways, either promoting rapid germination under severe water stress (*P. euryphylla*), delaying germination under no water stress ( *R. nudiflorum* ) or resulting in no significant changes (*P. bellidioides*). These contrasting responses are consistent with findings from arcto‐alpine species, where co‐occurring forbs or related graminoids show contrasting maternal effects on germination speed (Gya et al. 2023; Veselá et al. 2021). Such variation may reflect different ecological strategies, such as drought avoidance versus drought tolerance during early life‐history stages (Sumner and Venn 2021) or distinct evolutionary histories (Larson et al. 2020).

In alpine ecosystems where germination is already constrained, climatic factors can act to further restrict recruitment windows (Körner 2021; Walck et al. 2011), and maternal environmental effects may act as a short‐term buffer against increasing annual climate variability. As soil moisture deficits become more common (Hock et al. 2019; Sánchez‐Bayo and Green 2013), maternal effects may enhance germination success under drought, thereby increasing the chances of recruitment under unfavourable conditions. However, the species‐specific responses we found suggest that climate‐driven shifts in germination may alter community composition by favouring species with greater plasticity (Nicotra et al. 2010; Van Allen et al. 2021). For example, at our study sites, *P. euryphylla* may gain a competitive advantage due to enhanced germination under extreme drought (−1.0 MPa), while *P. bellidioides* may decline in abundance. Although germination responses may shape recruitment outcomes, their long‐term effects will ultimately depend on subsequent seedling survival and performance.

Finally, while our study focused on water stress responses under optimal thermal conditions for germination (20°C), future experiments incorporating a broader range of germination temperatures could help to evaluate the interactive effects of temperature and moisture, and improve predictions regarding the effects of climate change on germination traits (e.g., amount, timing and synchrony).

### 4.3 Seedling Performance

In this study, the drier maternal environment affected seedling morphological and physiological traits of our study species in different ways. The two forbs (*P. bellidioides* and *P. euryphylla*) responded similarly, showing clear maternal effects under severe water stress. In contrast, maternal effects in the graminoid *R. nudiflorum* were limited to morphological traits. One possible explanation is that *R. nudiflorum* may already possess physiological adaptations to water‐limited environments, which reduces the potential for additional maternal effects on physiological performance. This is supported by its consistently high relative water content, suggesting that it experienced less water stress than the forbs under the same conditions. This explanation also concurs with the previously reported differences in ecological strategies during early life‐history stages between forbs and graminoids, likely reflecting their different evolutionary histories (Larson et al. 2020). However, we are cautious to generalise since our data come from a single grass species. Nevertheless, our results suggest that pre‐existing drought tolerance may limit the extent of maternal effects on physiological traits while still influencing morphological adaptations.

In terms of morphological responses, seedlings from drier maternal environments developed greater total leaf area under drought conditions (60% pot capacity, although not significant for *P. bellidioides*). These results suggest that maternal effects can enhance offspring growth under low water availability conditions, which is consistent with findings from other systems, such as a temperate European annual forb (Sultan et al. 2009). However, our results also suggest a potential trade‐off: under well‐watered conditions, seedlings from drier maternal environments tended to be smaller, particularly for *P. bellidioides* and *P. euryphylla*, probably due to reduced seed size (see discussion of seed traits). Ecologically, this may place these species at a disadvantage in wet years preceded by dry maternal conditions, especially if favourable conditions trigger mass germination from the soil seed bank. Smaller seedlings might be outcompeted by larger conspecifics or individuals from other species, potentially reducing their chances of survival and establishment. This trade‐off was not observed in *R. nudiflorum* , where seedlings from the rainout treatment maintained higher TLA across all watering regimes (i.e., maternal effects expressed even in the absence of water stress, which could be potentially linked to conditions during seed development).

Physiological measurements taken at the end of the experiment further supported the presence of maternal effects in *P. euryphylla* and *P. bellidioides*, particularly under severe water stress. Although the measured variables represent different physiological processes (RWC reflecting structural‐hydraulic status and *F*_V_/*F*_M_ photochemical performance), their co‐expression in rainout seedlings suggests a coordinated stress response, characterised by improved water retention and enhanced photosynthetic function. Such responses are likely to be beneficial for early seedling establishment under drought. In contrast, *R. nudiflorum* showed no significant physiological differences between maternal environments, despite clear morphological differences, suggesting that maternal effects in this species have more influence on structural than on physiological adaptations.

Temporal analysis of *F*_V_/*F*_M_ trajectories over the course of the experiment revealed a transient dip in values on Day 7, likely reflecting the stress associated with transferring seedlings from the greenhouse to the growth chamber, where light intensity was lower. Interestingly, this unplanned stress treatment was less pronounced in seedlings from the rainout treatment, suggesting that maternal effects may not be limited to continuous stressors (e.g., water deficit), but may also enhance performance under co‐occurring or transient stress events.

Ecologically, these results suggest that maternal environmental effects may play an important role in alpine ecosystems, where newly germinated seedlings typically have poor tolerance to moisture stress (Mondoni et al. 2022; Vázquez‐Ramírez and Venn 2021). Although we did not assess other functional traits, such as specific leaf area (SLA) or root‐to‐shoot ratio, which could provide deeper insights into species‐specific strategies for drought adaptation (e.g., Gya et al. 2023; Harrison and LaForgia 2019; Larson et al. 2020), the observed correspondence between seed and seedling traits emphasises the importance of studying multiple life stages. A whole‐of‐life‐stage approach is essential for understanding how maternal effects can shape recruitment processes and predict population dynamics and community composition in alpine environments under climate change.

### 4.4 Implications for Conservation and Restoration of Alpine Environments

Our results suggest that by manipulating the maternal environment, it is possible to improve seed and seedling performance under predicted future climate conditions. Our methods are relevant for drought‐sensitive or range‐restricted alpine species where collecting ‘climate‐ready’ seeds from naturally adapted populations is not feasible (O'Neill and Gómez‐Pineda 2021; St. Clair et al. 2022).

In the Australian Alps, for example, many rare and threatened alpine plant species are restricted to summits (e.g., *Leucochrysum alpinum*) or specialised habitats such as wetlands (e.g., *Kelleria bogongensis*). These species often lack populations that span natural rainfall/moisture gradients, making it impossible to collect seeds from ‘dry‐adapted’ populations. In such cases, rainout shelters provide a practical solution for inducing drought‐related maternal effects, allowing the production of seeds and seedlings adapted to dry conditions. As we show here, this method can improve germination and seedling performance under water‐limited conditions, potentially increasing the success of restoration efforts.

Integrating maternal environmental manipulations into seed banking and restoration practices could complement existing genetic and provenance‐based strategies (Aitken and Whitlock 2013; Broadhurst et al. 2008; Ramalho et al. 2017) and improve the effectiveness of conservation and management actions under climate change. However, further research is needed to assess the long‐term fitness consequences of these maternal effects and to understand species‐specific responses in order to refine protocols for large‐scale seed production, restoration and conservation planning in alpine ecosystems.

## Author Contributions

**Jerónimo Vázquez‐Ramírez:** conceptualization (lead), data curation (lead), formal analysis (lead), funding acquisition (equal), investigation (lead), methodology (lead), writing – original draft (lead). **Daniel J. White:** investigation (equal), resources (equal), writing – review and editing (equal). **Judd Harding:** investigation (equal), resources (equal), writing – review and editing (equal). **Megan J. Hirst:** resources (equal), writing – review and editing (equal). **Tricia Wevill:** resources (equal), writing – review and editing (equal). **Susanna E. Venn:** funding acquisition (equal), investigation (equal), methodology (equal), resources (equal).

## Conflicts of Interest

The authors declare no conflicts of interest.

## Supporting information


**Appendix S1:** ece372247‐sup‐0001‐AppendixS1.docx.

## Data Availability

All data and R code used in this study are available for peer review at the following private Figshare link: https://figshare.com/s/dff649b3cdad28c1d88d. These materials will be published under a DOI via Figshare upon acceptance.
