# Supplementary material for: A Drier Maternal Environment Increases Water Stress Tolerance of Alpine Seeds and Seedlings
Source: Ecol Evol. 2025 Sep 29;15(10):e72247. doi: 10.1002/ece3.72247 (PMC12479109; doi:10.1002/ece3.72247)
Supplement: Supplementary file 1 — Appendix S1: ece372247‐sup‐0001‐AppendixS1.docx. [file ECE3-15-e72247-s001.docx]

**Supplementary materials**

**A drier maternal environment increases water stress tolerance of alpine seeds and seedlings**

The following supplementary material are available:

**Tables**

- **Table S1**. Results of linear model contrasting soil volumetric water content.
- **Table S2**. Results of linear models contrasting seed morphological traits.
- **Table S3**. Results of GAMM smooth terms for FPG and MGT
- **Table S4**. Results of GLMM fixed effects for FPG and MGT
- **Table S5**. Results of FPG and MGT pairwise comparisons
- **Table S6**. Results of GLMM fixed effects for TLA, RWC and F_V_/F_M_
- **Table S7**. Results of TLA, RWC and F_V_/F_M_ pairwise comparisons
- **Table S8**. Results of weekly F_V_/F_M_ values pairwise comparisons
- **Table S9.** Results of LRT between full and reduced GAMM models for weekly F_V_/F_M_ values

**Figures**

- **Figure S1**. Mean soil volumetric water content in control and sheltered plots

**Table S1.** Results of the robust linear mixed model contrasting soil volumetric water content in shelter and control plots

|  | **Estimate** | **Std. Error** | **t-value** | **p-value** |
| --- | --- | --- | --- | --- |
| **Control** | 0.325 | 0.002 | 160.91 | <0.001 |
| **Shelter** | -0.038 | 0.002 | -13.18 | <0.001 |

**Table S2.** Results of linear models contrasting 100-seed mass (g) and seed length (cm) during the seed developing stage in control and rain-out shelter conditions for the study species. *Log-transformed data

| **Species** | **Condition** | **Estimate** | **Std. Error** | **t-value** | **p-value** |
| --- | --- | --- | --- | --- | --- |
| **100-Seed mass** | | | | | |
| *P. bellidioides* | Control | 0.038 | 0.001 | 84.97 | <0.001 |
|  | Shelter | -0.002 | 0.001 | -3.16 | 0.013 |
| *P. euryphylla** | Control | -2.496 | 0.018 | -134.32 | <0.001 |
|  | Shelter | -0.058 | 0.026 | -2.22 | 0.046 |
| *R. nudiflorum* | Control | 0.039 | 0.001 | 68.94 | <0.001 |
|  | Shelter | 0.006 | 0.001 | 7.75 | <0.001 |
| Seed length | | | | | |
| *P. bellidioides* | Control | 0.377 | 0.005 | 69.52 | <0.001 |
|  | Shelter | -0.036 | 0.007 | -4.78 | <0.001 |
| *P. euryphylla* | Control | 0.154 | 0.003 | 43.76 | <0.001 |
|  | Shelter | -0.014 | 0.004 | -2.93 | <0.009 |
| *R. nudiflorum* | Control | 0.191 | 0.005 | 37.69 | <0.001 |
|  | Shelter | 0.013 | 0.007 | 1.931 | 0.069 |

**Table S3.** Summary of GAMM smooth terms for Final Germination Percentage and Mean Germination Rate

| **Species** | **Condition** | **edf** | **F** | **p-value** |
| --- | --- | --- | --- | --- |
| **Final Germination Percentage** | | | | |
| *P. euryphylla* | Control | 1.0 | 21.75 | <0.001 |
|  | Shelter | 1.0 | 0.26 | 0.61 |
| *P. bellidioides* | Control | 1.0 | 30.95 | <0.001 |
|  | Shelter | 1.0 | 8.27 | 0.006 |
| *R. nudiflorum* | Control | 1.6 | 9.38 | 0.008 |
|  | Shelter | 1.0 | 0.28 | 0.60 |
| **Mean Germination Rate** | | | | |
| *P. bellidioides* | Control | 1.0 | 12.779 | 0.001 |
|  | Shelter | 1.0 | 7.099 | 0.010 |
| *P. euryphylla* | Control | 1.43 | 1.973 | 0.311 |
|  | Shelter | 1.73 | 1.479 | 0.290 |
| *R. nudiflorum* | Control | 1.48 | 2.327 | 0.067 |
|  | Shelter | 1.000 | 1.339 | 0.253 |

**Table S4**. Summary of GLMM fixed effects for Final Germination Percentage and Mean Germination Rate

| **Species** | **Effect** | **Estimate** | **Std. Error** | **p-value** |
| --- | --- | --- | --- | --- |
| **Final Germination Percentage** | | | | |
| *P. bellidioides* | Intercept | 0.08 | 0.2 | 0.689 |
|  | Shelter | 0.58 | 0.29 | 0.045 |
|  | Ψ = -0.75 | 0.54 | 0.29 | 0.063 |
|  | Ψ = -0.5 | 1.31 | 0.32 | <0.001 |
|  | Ψ = 0 | 1.82 | 0.36 | <0.001 |
| *P. euryphylla* | Intercept | 1.27 | 0.24 | <0.001 |
|  | Shelter | 1.32 | 0.46 | 0.004 |
|  | Ψ = -0.75 | 1.32 | 0.46 | 0.004 |
|  | Ψ = -0.5 | 1.18 | 0.44 | 0.008 |
|  | Ψ = 0 | 2.63 | 0.75 | <0.001 |
| *R. nudiflorum* | Intercept | 0.58 | 0.32 | 0.064 |
|  | Shelter | 1.64 | 0.56 | 0.003 |
|  | Ψ = -0.75 | 1.09 | 0.49 | 0.025 |
|  | Ψ = -0.5 | 1.43 | 0.53 | 0.007 |
|  | Ψ = 0 | 1.25 | 0.51 | 0.013 |
| **Mean Germination Rate** | | | | |
| *P. bellidioides* | Intercept | 0.053 | 0.004 | <0.001 |
|  | Shelter | 0.004 | 0.006 | 0.466 |
|  | Ψ = -0.75 | 0.006 | 0.006 | 0.378 |
|  | Ψ = -0.5 | 0.022 | 0.006 | 0.002 |
|  | Ψ = 0 | 0.024 | 0.006 | <0.001 |
| *P. euryphylla* | Intercept | 0.121 | 0.005 | <0.001 |
|  | Shelter | -0.023 | 0.007 | 0.002 |
|  | Ψ = -0.75 | -0.008 | 0.007 | 0.268 |
|  | Ψ = -0.5 | -0.012 | 0.007 | 0.103 |
|  | Ψ = 0 | -0.005 | 0.007 | 0.481 |
| *R. nudiflorum* | Intercept | 0.139 | 0.007 | 0.002 |
|  | Shelter | 0.001 | 0.010 | 0.926 |
|  | Ψ = -0.75 | 0.001 | 0.010 | 0.911 |
|  | Ψ = -0.5 | 0.006 | 0.010 | 0.524 |
|  | Ψ = 0 | -0.022 | 0.010 | 0.028 |

**Table S5.** Results of pairwise comparisons contrasting Final Germination Percentage and Mean Germination Rate (Dry Maternal Environment - Control)

| **Final Germination Percentage** | | | | | |
| --- | --- | --- | --- | --- | --- |
| **Water Potential** | **Odds. Ratio** | **Std. Error** | **df** | **z-ratio** | **p-value** |
| *Pappochroma bellidioides* | | | | | |
| 0 | 1.181 | 0.482 | Inf | 0.407 | 0.683 |
| -0.25 | 1 | 0.354 | Inf | 0 | 1 |
| -0.05 | 0.85 | 0.28 | Inf | -0.493 | 0.622 |
| -0.75 | 0.619 | 0.193 | Inf | -1.538 | 0.124 |
| -1 | 0.558 | 0.162 | Inf | -2.005 | **0.045** |
| *Plantago euryphylla* | | | | | |
| 0 | 2.042 | 1.79 | Inf | 0.813 | 0.416 |
| -0.25 | 0.866 | 0.466 | Inf | -0.268 | 0.788 |
| -0.05 | 0.356 | 0.246 | Inf | -1.493 | 0.135 |
| -0.75 | 0.699 | 0.422 | Inf | -0.593 | 0.553 |
| -1 | 0.267 | 0.123 | Inf | -2.87 | **0.004** |
| *Rytidosperma nudiflorum* | | | | | |
| 0 | 0.534 | 0.353 | Inf | -0.948 | 0.343 |
| -0.25 | 0.638 | 0.433 | Inf | -0.663 | 0.507 |
| -0.05 | 0.638 | 0.433 | Inf | -0.663 | 0.507 |
| -0.75 | 0.716 | 0.416 | Inf | -0.575 | 0.565 |
| -1 | 0.198 | 0.11 | Inf | -2.918 | **0.003** |
| **Mean Germination Rate** | | | | | |
| **Water potential** | **Estimate** | **Std. Error** | **df** | **t-ratio** | **p-value** |
| *Pappochroma bellidioides* | | | | | |
| 0 | -0.003 | 0.006 | 36 | 0.547 | 0.587 |
| -0.25 | 0.004 | 0.006 | 36 | -0.647 | 0.521 |
| -0.05 | -0.009 | 0.006 | 36 | 1.459 | 0.153 |
| -0.75 | 0.001 | 0.006 | 36 | -0.1 | 0.921 |
| -1 | 0.004 | 0.006 | 36 | -0.736 | 0.466 |
| *Plantago euryphylla* | | | | | |
| 0 | 0.013 | 0.007 | 36 | 1.787 | 0.082 |
| -0.25 | -0.003 | 0.007 | 36 | -0.42 | 0.676 |
| -0.05 | -0.004 | 0.007 | 36 | -0.536 | 0.595 |
| -0.75 | 0.001 | 0.007 | 36 | 0.161 | 0.872 |
| -1 | 0.023 | 0.007 | 36 | 3.183 | **0.003** |
| *Rytidosperma nudiflorum* | | | | | |
| 0 | 0.030 | 0.01 | 36 | -3.035 | **0.004** |
| -0.25 | 0.022 | 0.01 | 36 | -2.222 | **0.032** |
| -0.05 | -0.0184 | 0.01 | 36 | 1.832 | 0.075 |
| -0.75 | -0.003 | 0.01 | 36 | -0.337 | 0.737 |
| -1 | -0.00093 | 0.01 | 36 | -0.093 | 0.9268 |

**Table S6**. Summary of GLMM fixed effects for total leaf area, relative water content and chlorophyll fluorescence including fixed effect estimates and interaction terms for each species.

| **Total Leaf Area** | | | | |
| --- | --- | --- | --- | --- |
| **Term** | **Estimate** | **Std. Error** | **t-value** | **p-value** |
| *Pappochroma bellidioides* | | | | |
| (Intercept) | 38.220 | 2.629 | 14.541 | 0.002 |
| Rainout shelter | -4.076 | 3.717 | -1.096 | 0.284 |
| Water 80% | 2.021 | 3.717 | 0.544 | 0.592 |
| Water 100% | 17.128 | 3.717 | 4.608 | <0.001 |
| Rainout:Water 80% | 5.134 | 5.257 | 0.976 | 0.339 |
| Rainout:Water 100% | -9.795 | 5.257 | -1.863 | 0.075 |
| *Plantago euryphylla* | | | | |
| (Intercept) | 1.260 | 0.204 | 6.153 | <0.001 |
| Rainout shelter | 1.482 | 0.289 | 5.115 | <0.001 |
| Water 80% | 1.743 | 0.289 | 6.017 | <0.001 |
| Water 100% | 4.856 | 0.289 | 16.757 | <0.001 |
| Rainout:Water 80% | -0.890 | 0.409 | -2.173 | 0.039 |
| Rainout:Water 100% | -2.265 | 0.409 | -5.528 | <0.001 |
| *Rytidosperma nudiflorum* | | | | |
| (Intercept) | 0.966 | 0.525 | 1.841 | 0.078 |
| Rainout shelter | 2.536 | 0.743 | 3.416 | 0.002 |
| Water 80% | -0.087 | 0.743 | -0.117 | 0.908 |
| Water 100% | 0.078 | 0.743 | 0.105 | 0.917 |
| Rainout:Water 80% | 0.903 | 1.05 | 0.859 | 0.399 |
| Rainout:Water 100% | 3.914 | 1.05 | 3.727 | 0.001 |
| **Relative Water Content** | | | | |
| *Pappochroma bellidioides* | | | | |
| (Intercept) | 84.303 | 2.134 | 39.512 | <0.001 |
| Rainout shelter | 6.476 | 3.017 | 2.146 | 0.042 |
| Water 80% | 1.011 | 3.017 | 0.335 | 0.740 |
| Water 100% | 4.686 | 3.017 | 1.553 | 0.133 |
| Rainout:Water 80% | -1.574 | 4.267 | -0.369 | 0.715 |
| Rainout:Water 100% | -3.105 | 4.267 | -0.728 | 0.473 |
| *Plantago euryphylla* | | | | |
| (Intercept) | 87.302 | 1.184 | 73.699 | <0.001 |
| Rainout shelter | 3.502 | 1.675 | 2.09 | 0.047 |
| Water 80% | 0.949 | 1.675 | 0.567 | 0.576 |
| Water 100% | 4.126 | 1.675 | 2.463 | 0.021 |
| Rainout:Water 80% | -2.422 | 2.369 | -1.022 | 0.316 |
| Rainout:Water 100% | -2.785 | 2.369 | -1.176 | 0.251 |
| *Rytidosperma nudiflorum* | | | | |
| (Intercept) | 87.322 | 1.808 | 48.3 | <0.001 |
| Rainout shelter | 4.461 | 2.712 | 1.645 | 0.114 |
| Water 80% | 3.538 | 2.557 | 1.384 | 0.180 |
| Water 100% | 7.798 | 2.712 | 2.876 | 0.008 |
| Rainout:Water 80% | -1.764 | 3.727 | -0.473 | 0.640 |
| Rainout:Water 100% | -1.5 | 3.835 | -0.391 | 0.699 |
| **F_V_/F_M_** | | | | |
| *Pappochroma bellidioides* | | | | |
| (Intercept) | 0.720 | 0.010 | 71.732 | <0.001 |
| Rainout shelter | 0.032 | 0.014 | 2.296 | 0.024 |
| Water 80% | 0.061 | 0.014 | 4.324 | <0.001 |
| Water 100% | 0.063 | 0.014 | 4.465 | <0.001 |
| Rainout:Water 80% | -0.020 | 0.020 | -1.019 | 0.311 |
| Rainout:Water 100% | -0.024 | 0.020 | -1.205 | 0.231 |
| *Plantago euryphylla* | | | | |
| (Intercept) | 0.766 | 0.009 | 80.484 | <0.001 |
| Rainout shelter | 0.055 | 0.013 | 4.15 | <0.001 |
| Water 80% | 0.049 | 0.013 | 3.705 | <0.001 |
| Water 100% | 0.059 | 0.013 | 4.428 | <0.001 |
| Rainout:Water 80% | -0.058 | 0.019 | -3.057 | 0.002 |
| Rainout:Water 100% | -0.060 | 0.019 | -3.166 | 0.002 |
| *Rytidosperma nudiflorum* | | | | |
| (Intercept) | 0.725 | 0.008 | 87.209 | <0.001 |
| Rainout shelter | 0.007 | 0.011 | 0.635 | 0.527 |
| Water 80% | 0.024 | 0.011 | 2.051 | 0.043 |
| Water 100% | 0.042 | 0.011 | 3.598 | <0.001 |
| Rainout:Water 80% | 0.025 | 0.016 | 1.543 | 0.126 |
| Rainout:Water 100% | 0.012 | 0.016 | 0.725 | 0.470 |

**Table S7**. Pairwise comparisons contrasting TLA, RWC, F_V_/F_M_, between maternal environments (Control vs. Rainout shelter) within each watering treatment for each species.

| **Total Leaf Area (TLA)** | | | | | | | | | | | | | | | |
| --- | --- | --- | --- | --- | --- | --- | --- | --- | --- | --- | --- | --- | --- | --- | --- |
| **Treatment** | **Estimate** | | | **Std. Error** | | | **df** | | | **t-ratio** | | | **p-value** | | |
| *Pappochroma bellidioides* | | | | | | | | | | | | | | | |
| 60% | 4.08 | | | 3.72 | | | 24 | | | 1.096 | | | 0.283 | | |
| 80% | -1.06 | | | 3.72 | | | 24 | | | -0.284 | | | 0.778 | | |
| 100% | 13.87 | | | 3.72 | | | 24 | | | 3.731 | | | 0.001 | | |
| *Plantago euryphylla* | | | | | | | | | | | | | | | |
| 60% | -1.482 | | | 0.29 | | | 24 | | | -5.115 | | | <.0001 | | |
| 80% | -0.592 | | | 0.29 | | | 24 | | | -2.042 | | | 0.052 | | |
| 100% | 0.783 | | | 0.29 | | | 24 | | | 2.702 | | | 0.012 | | |
| *Rytidosperma nudiflorum* | | | | | | | | | | | | | | | |
| 60% | -2.54 | | | 0.743 | | | 24 | | | -3.416 | | | 0.002 | | |
| 80% | -3.44 | | | 0.743 | | | 24 | | | -4.631 | | | 0.001 | | |
| 100% | -6.45 | | | 0.743 | | | 24 | | | -8.686 | | | <0.001 | | |
| **Relative Water Content (RWC)** | | | | | | | | | | | | | | | |
| *Pappochroma bellidioides* | | | | | | | | | | | | | | | |
| 60% | | | -6.48 | | | 3.02 | | | 24 | | | -2.146 | | | 0.042 |
| 80% | | | -4.9 | | | 3.02 | | | 24 | | | -1.625 | | | 0.117 |
| 100% | | | -3.37 | | | 3.02 | | | 24 | | | -1.117 | | | 0.275 |
| *Plantago euryphylla* | | | | | | | | | | | | | | | |
| 60% | -3.502 | | | 1.68 | | | 24 | | | -2.09 | | | 0.047 | | |
| 80% | -1.08 | | | 1.68 | | | 24 | | | -0.645 | | | 0.525 | | |
| 100% | -0.716 | | | 1.68 | | | 24 | | | -0.428 | | | 0.672 | | |
| *Rytidosperma nudiflorum* | | | | | | | | | | | | | | | |
| 60% | -4.46 | | | 2.71 | | | 22 | | | -1.645 | | | 0.114 | | |
| 80% | -2.7 | | | 2.56 | | | 22 | | | -1.055 | | | 0.302 | | |
| 100% | -2.96 | | | 2.71 | | | 22 | | | -1.092 | | | 0.286 | | |
| **F_V_/F_M_** | | | | | | | | | | | | | | | |
| *Pappochroma bellidioides* | | | | | | | | | | | | | | | |
| 60% | | -0.032 | | | 0.014 | | | 84 | | | -2.296 | | | 0.024 | |
| 80% | | -0.012 | | | 0.014 | | | 84 | | | -0.854 | | | 0.395 | |
| 100% | | -0.008 | | | 0.014 | | | 84 | | | -0.592 | | | 0.555 | |
| *Plantago euryphylla* | | | | | | | | | | | | | | | |
| 60% | | -0.055 | | | 0.013 | | | 84 | | | -4.15 | | | <0.001 | |
| 80% | | 0.002 | | | 0.013 | | | 84 | | | 0.173 | | | 0.862 | |
| 100% | | 0.004 | | | 0.013 | | | 84 | | | 0.327 | | | 0.744 | |
| *Rytidosperma nudiflorum* | | | | | | | | | | | | | | | |
| 60% | | -0.007 | | | 0.011 | | | 84 | | | -0.635 | | | 0.527 | |
| 80% | | -0.033 | | | 0.011 | | | 84 | | | -2.816 | | | 0.061 | |
| 100% | | -0.019 | | | 0.011 | | | 84 | | | -1.66 | | | 0.100 | |

**Table S8**. Results of the F_V_/F_M_ pairwise comparison between seedlings grown from seed of control and shelter plots at the 3 measured times.

| **Watering** | **Week** | **Estimate** | **Std. Error** | **df** | **t-ratio** | **p-value** |  | |
| --- | --- | --- | --- | --- | --- | --- | --- | --- |
| *Pappochroma bellidioides* | | | | | | | |  |
| 100% | 0 | -0.002 | 0.012 | 84.1 | -0.226 | 0.821 |  | |
|  | 2 | -0.013 | 0.012 | 84.1 | -1.093 | 0.277 |  | |
|  | 4 | -0.008 | 0.012 | 84.1 | -0.688 | 0.493 |  | |
| 80% | 0 | 0.013 | 0.015 | 84.2 | 0.893 | 0.374 |  | |
|  | 2 | -0.033 | 0.014 | 84.2 | -2.254 | 0.026 |  | |
|  | 4 | -0.011 | 0.015 | 84.2 | -0.762 | 0.447 |  | |
| 60% | 0 | -0.002 | 0.016 | 84.2 | -0.13 | 0.897 |  | |
|  | 2 | -0.039 | 0.015 | 84.2 | -2.519 | 0.013 |  | |
|  | 4 | -0.026 | 0.016 | 84.2 | -1.649 | 0.029 |  | |
| *Plantago euryphylla* | | | | | | | |  |
| 100% | 0 | 0.009 | 0.011 | 84.1 | 0.813 | 0.418 |  | |
|  | 2 | -0.030 | 0.011 | 84.1 | -2.683 | 0.008 |  | |
|  | 4 | 0.027 | 0.011 | 84.1 | 2.382 | 0.019 |  | |
| 80% | 0 | -0.006 | 0.011 | 84.0 | -0.57 | 0.570 |  | |
|  | 2 | -0.028 | 0.010 | 84.0 | -2.654 | 0.009 |  | |
|  | 4 | 0.002 | 0.011 | 84.0 | 0.227 | 0.821 |  | |
| 60% | 0 | 0.012 | 0.017 | 84.4 | 0.688 | 0.493 |  | |
|  | 2 | -0.025 | 0.016 | 84.4 | -1.529 | 0.129 |  | |
|  | 4 | -0.055 | 0.017 | 84.4 | -3.193 | 0.002 |  | |
| *Rytidosperma nudiflorum* | | | | | | | |  |
| 100% | 0 | -0.008 | 0.017 | 84.2 | -0.45 | 0.653 |  | |
|  | 2 | -0.014 | 0.017 | 84.2 | -0.837 | 0.405 |  | |
|  | 4 | -0.019 | 0.017 | 84.2 | -1.092 | 0.277 |  | |
| 80% | 0 | 0.004 | 0.016 | 85.0 | 0.291 | 0.772 |  | |
|  | 2 | -0.008 | 0.013 | 85.0 | -0.616 | 0.539 |  | |
|  | 4 | -0.037 | 0.016 | 85.0 | -2.304 | 0.053 |  | |
| 60% | 0 | -0.010 | 0.014 | 84.3 | -0.68 | 0.498 |  | |
|  | 2 | -0.018 | 0.014 | 84.3 | -1.26 | 0.211 |  | |
|  | 4 | -0.007 | 0.014 | 84.3 | -0.474 | 0.636 |  | |

**Table S9**. Results of LRT test between full and reduced (no interaction between water potential and maternal environment) GAMMS models of weekly F_V_/F_M_ values

| **Species** | **Watering** | **L.Ratio** | **p-value** |
| --- | --- | --- | --- |
| *P. bellidioides* | 100% | 3.42 | 0.180 |
|  | 80% | 2.17 | 0.338 |
|  | 60% | 0.05 | 0.977 |
| *P. euryphylla* | 100% | 8.75 | 0.012 |
|  | 80% | 1.4 | 0.497 |
|  | 60% | 6.47 | 0.039 |
| *R. nudiflorum* | 100% | 2.61 | 0.271 |
|  | 80% | 2.24 | 0.326 |
|  | 60% | 2.05 | 0.358 |


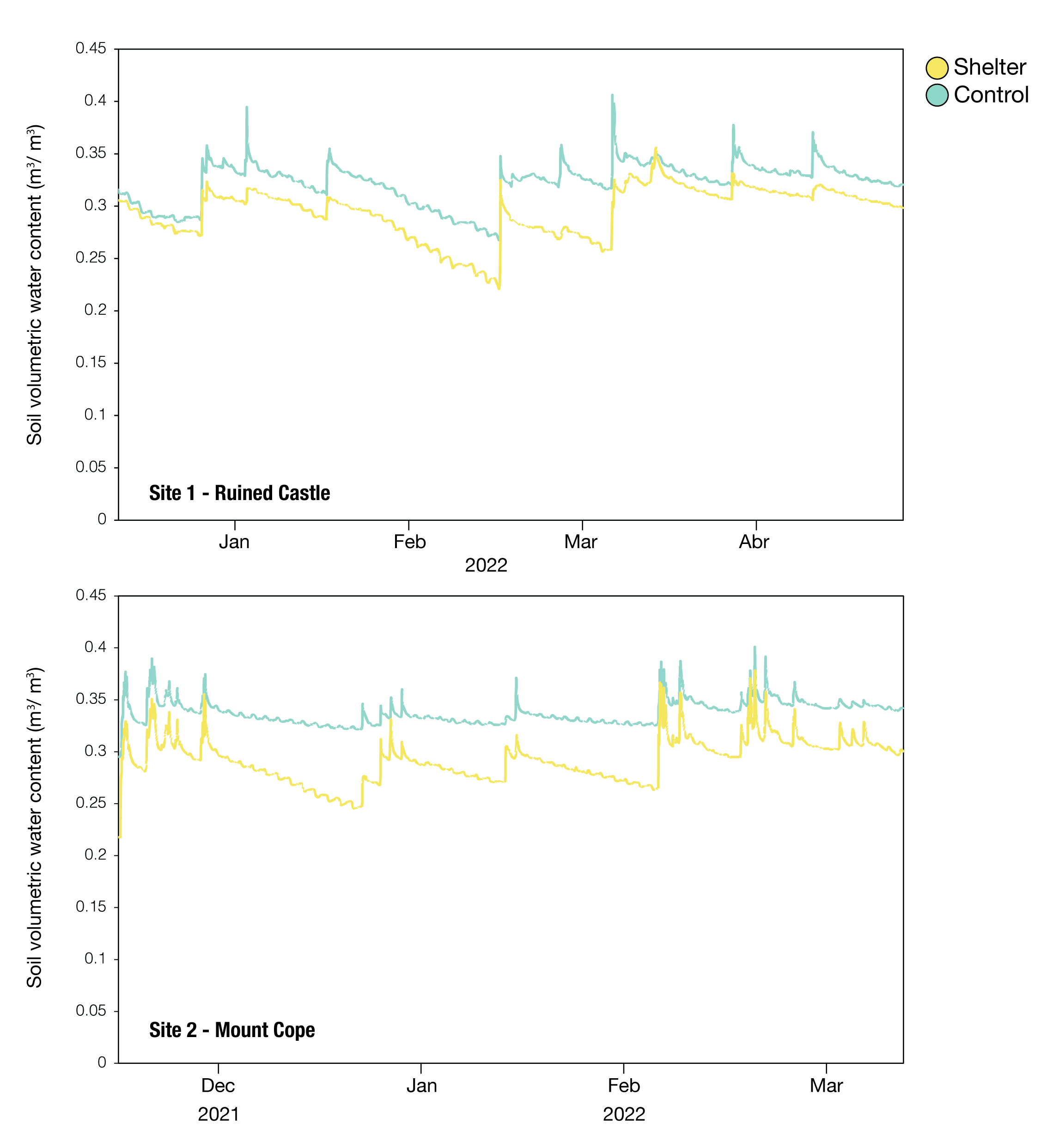


**Figure S1.** Mean soil volumetric water content in control and sheltered plots at the two experimental sites during part of the seed development stage (see Figure for their location) Due to micro-stations and sensor malfunctions the periods shown vary between sites and do not represent the full snow-free season (Nov-May)
